# Editorial on hypothesis and objectives in clinical trials: superiority, equivalence and non-inferiority

**DOI:** 10.1186/1477-9560-7-3

**Published:** 2009-03-21

**Authors:** Claudio D Gonzalez, Ricardo Bolaños, Martha de Sereday

**Affiliations:** 1 Department of Pharmacology, School of Medicine, University of Buenos Aires, Buenos Aires, Argentina; 2 Committee of Epidemiology, Argentinean Society of Diabetes, Buenos Aires, Argentina

## Editorial

Randomized clinical trial is often considered as the Gold Standard method for comparing treatment effects. In practice, taking into consideration their main objectives, the majority of clinical trials are aimed to establish the superiority of an intervention regarding to an active control or placebo [1]. Within the methodological core of these so called *superiority trials*, the assessment of the statistical signification of the differences between or among interventions, and their clinical relevance, are both of main importance. Appropriate statistical tests to assess this superiority should be performed, with the *null hypothesis *being: *the difference between treatments is equal to 0 (H0 ≡ Δ = 0)*, and the alternative hypothesis: *treatments are different -or, the difference between treatments is not equal to 0 (H1 ≡ Δ ≠ 0)(if two sided)-*. The rejection of the null hypothesis is in the foundation of the methodological assessment of superiority [2]. The number of patients required to confront the hypotheses is inversely related to the expected between-treatment differences. The smallest the expected difference between two interventions, the highest the number of patients to be included into the trial. But, how to interpret a non-significant result obtained from a clinical study designed as a superiority trial? Does this mean that the interventions under study should be considered as *equivalent*?. Clearly, the answer is negative. From the methodological standpoint, the expression: *we have no evidence of difference between treatments*, should not be considered as equivalent to: *we have evidence of no difference between interventions *[3].

Very often, the aim of a clinical trial is to show that a certain intervention is equivalent to or non inferior than another one. In this case, as stated before, a non-significant superiority testing should not be interpreted as a proof of no difference between treatments. Under an equivalence hypothesis, where the between treatments difference is assumed to be equal to 0, the calculation of a sample size following the rules established for superiority trials is impossible or in the best case (by employing an estimated difference close to 0) would result in an unrealistic extremely large number [1]. Instead, equivalence and non-inferiority trials should be conceived, planned and applied to these purposes. In general, neither equivalence nor non-inferiority should be definitively concluded from superiority trials exhibiting non-significant results.

Improvements into the galenics of a formulation or a modification in a drug delivery system should not affect the pharmacokinetic (PK) profile of the drug. That is also the case for the comparison between the PK parameters of a generic preparation versus an original product. This is the rationale for the so called *bioequivalence trials*, the most frequent type of equivalence study. Being μ_S _the mean value for an specific PK indicator in the *standard *or the original product, and μ_T _the mean for the same parameter of the new product under testing, the null hypothesis in a bioequivalence trial would be: H0 ≡ μ_S_-μ_T _≤ -Δ or μ_S_-μ_T_>Δ, (where Δ is the magnitude of a prespecified difference between the standard (S) and the new product T. Typically, the main PK indicators under study are: the peak concentration of the drugs on trial (Cmax), their areas-under-the-curve of concentrations in relation to time (AUC) and the time-to-peak (Tmax) [4].

The purpose of a "non-inferiority trial" is to show that a new intervention is no worse than the standard reference therapy. Here, the assumption is that the difference in effect is no less than a prespecified Δ. It should be designed as a one-sided trial. The *null hypothesis *is: H0 ≡ π_S_-π_T _≥ M, being π_S _and π_T _the outcomes rate for a qualitative dependent variable (e.g. death, infarctions, stroke), and M a maximum allowable limit of difference, or, H0 ≡ μ_S_-μ_T _≥ M in case of a quantitative outcome (where μ are means).

Non-inferiority trials are carried out when: 1) a placebo-controlled trial is not ethically feasible; 2) the treatment under test is not expected to be better that the standard or reference intervention in terms of efficacy, but is supposedly better regarding to other secondary endpoints, safety, costs, compliance or convenience [2].

In non-inferiority trials, the choice of the maximum allowable margin is crucial. The rationale to determine these limits require careful consideration and should be appropriately described into the specific section of the protocol. ASSENT 2 trial is an early example of non-inferiority trial within the scope of thrombolytic therapy [5,6]. Alternative hypothesis in this study was that the 30 days mortality is not inferior for a single bolus of tecneplase when compared with an accelerate infusion of alteplase. The path to determine the allowable margin involved several steps. It was finally accepted that considering an event rate cut off of 7.2% in patients treated with alteplase, the non-inferiority area could be defined as follows: if π_S _(alteplase events rate) were >7.2%, then π_S_-π_T _(difference in events between alteplase and tecneplase) <1%; in case of a π_S _< 7.2%, a ratio π_S_/π_T _< 1.14 would be considered as within the non-inferiority region. Results of this trial showed that the events rate for the alteplase treated group was 6.18%; in the tecneplase treated patients was 6.16%. Events rate in the alteplase group was below 7.2; as the ratio π_S_/π_T _was 0.99 (with a 90% confidence interval of 0.90 to 1.10) always below 1.14 (even considering the upper 90% confidence interval limit), the non-inferiority hypothesis was considered as proven. TARGET (Do Tirofiban and ReoPro Give Similar Efficacy Outcomes Trial) is another good example of non-inferiority trial, comparing two glycoprotein IIb/IIIa receptor blockers [7]; nevertheless, in this case, tirofiban failed to demonstrate non-inferiority to abciximab (the ischemic event rate was higher in the tirofiban group).

Non-inferiority trials offer some issues to the methodological debate: selection of the appropriate margin could be sometimes considered as arbitrary (it is dependant on the indication, the clinical judgment and the regulatory guidance), and in many occasions would require a clear estimation/prediction of the effect of the control therapy (and this is not always available). While in superiority trials *intention-to-treat *(ITT) based analysis is always recommendable, it is unclear that this approach could provide any benefits in non-inferiority trials and "*per procolol*" analyses are preferred by some authors. In the ITT approach, all patients we intended to treat will be included into the analysis, whether they completed the trial following the protocol or not. The purpose is to minimize the potential impact of withdrawals and non-compliance on the interpretation of the results. Conversely, in a "as per protocol" breakdown, patients who were non-compliant, or unable to complete the assigned treatment or have missing data are excluded from the analysis. In a pragmatic approach, most of the specialists recommend to apply both ITT and "per protocol" analyses, assuring that the results of these two analysis are not conflictive. Nevertheless, in the field of non-inferiority trials, this issue is still a matter of methodological controversy [2,8].

In spite of the limitations in the doctrine that supports the classification of the clinical experiments as "superiority", "equivalence" and "non-inferiority" trials, this methodological approach allowed for a improved production and understanding of the medical evidence when compared with the traditional view. Further epistemological refinements of this theory are granted.
